# Secondary prevention of Alzheimer’s dementia: neuroimaging contributions

**DOI:** 10.1186/s13195-018-0438-z

**Published:** 2018-10-30

**Authors:** Mara ten Kate, Silvia Ingala, Adam J. Schwarz, Nick C. Fox, Gaël Chételat, Bart N. M. van Berckel, Michael Ewers, Christopher Foley, Juan Domingo Gispert, Derek Hill, Michael C. Irizarry, Adriaan A. Lammertsma, José Luis Molinuevo, Craig Ritchie, Philip Scheltens, Mark E. Schmidt, Pieter Jelle Visser, Adam Waldman, Joanna Wardlaw, Sven Haller, Frederik Barkhof

**Affiliations:** 1 Department of Radiology and Nuclear Medicine, Neuroscience Campus Amsterdam, VU University Medical Center, Amsterdam, the Netherlands; 2 Alzheimer Center & Department of Neurology, Neuroscience Campus Amsterdam, VU University Medical Center, PO Box 7056, 1007 MB Amsterdam, the Netherlands; 3 Takeda Pharmaceuticals Comparny, Cambridge, MA USA; 4 Eli Lilly and Company, Indianapolis, Indiana USA; 5 Dementia Research Centre, Department of Neurodegenerative Disease, UCL Institute of Neurology, London, UK; 6 Institut National de la Santé et de la Recherche Médicale, Inserm UMR-S U1237, Université de Caen-Normandie, GIP Cyceron, Caen, France; 7 Institute for Stroke and Dementia Research, Klinikum der Universität München, Ludwig-Maximilians-Universität LMU, Munich, Germany; 8 GE Healthcare Life Sciences, Amersham, UK; 9 Barcelonaβeta Brain Research Center, Pasqual Maragall Foundation, Barcelona, Spain; 10 IXICO Plc, London, UK; 11 Centre for Dementia Prevention, Centre for Clinical Brain Sciences, University of Edinburgh, Edinburgh, UK; 12 Janssen Pharmaceutica NV, Beerse, Belgium; 13 Dementia Research Centre, University of Edinburgh, Edinburgh, UK; 14 Affidea Centre de Diagnostic Radiologique de Carouge, Geneva, Switzerland; 15 Insititutes of Neurology and Healthcare Engineering, University College London, London, UK

**Keywords:** Alzheimer’s disease, Neuroimaging, Secondary prevention, Clinical trials

## Abstract

**Background:**

In Alzheimer’s disease (AD), pathological changes may arise up to 20 years before the onset of dementia. This pre-dementia window provides a unique opportunity for secondary prevention. However, exposing non-demented subjects to putative therapies requires reliable biomarkers for subject selection, stratification, and monitoring of treatment. Neuroimaging allows the detection of early pathological changes, and longitudinal imaging can assess the effect of interventions on markers of molecular pathology and rates of neurodegeneration. This is of particular importance in pre-dementia AD trials, where clinical outcomes have a limited ability to detect treatment effects within the typical time frame of a clinical trial. We review available evidence for the use of neuroimaging in clinical trials in pre-dementia AD. We appraise currently available imaging markers for subject selection, stratification, outcome measures, and safety in the context of such populations.

**Main body:**

Amyloid positron emission tomography (PET) is a validated in-vivo marker of fibrillar amyloid plaques. It is appropriate for inclusion in trials targeting the amyloid pathway, as well as to monitor treatment target engagement. Amyloid PET, however, has limited ability to stage the disease and does not perform well as a prognostic marker within the time frame of a pre-dementia AD trial. Structural magnetic resonance imaging (MRI), providing markers of neurodegeneration, can improve the identification of subjects at risk of imminent decline and hence play a role in subject inclusion. Atrophy rates (either hippocampal or whole brain), which can be reliably derived from structural MRI, are useful in tracking disease progression and have the potential to serve as outcome measures. MRI can also be used to assess comorbid vascular pathology and define homogeneous groups for inclusion or for subject stratification. Finally, MRI also plays an important role in trial safety monitoring, particularly the identification of amyloid-related imaging abnormalities (ARIA). Tau PET to measure neurofibrillary tangle burden is currently under development. Evidence to support the use of advanced MRI markers such as resting-state functional MRI, arterial spin labelling, and diffusion tensor imaging in pre-dementia AD is preliminary and requires further validation.

**Conclusion:**

We propose a strategy for longitudinal imaging to track early signs of AD including quantitative amyloid PET and yearly multiparametric MRI.

**Electronic supplementary material:**

The online version of this article (10.1186/s13195-018-0438-z) contains supplementary material, which is available to authorized users.

## Background

Alzheimer’s disease (AD) is the leading cause of dementia worldwide and it is pathologically characterised by the deposition of extracellular β-amyloid plaques and intracellular neurofibrillary tangles of hyperphosphorylated tau proteins [1]. Pathological changes may start up to 20 years before the onset of symptoms [2–5]. To date, treatments have only been approved for the dementia stage of the disease and provide modest symptomatic benefit but no slowing of progression. A global research priority is to find therapies to prevent or delay symptom onset and functional decline [6], and a number of candidate agents have shown promise in cell or animal models. Despite this, clinical trials of potential disease-modifying treatments have proven unsuccessful thus far. This may be partly due to limitations of the treatments (e.g. incorrect molecular target or inadequate target engagement, dose, or duration). However, the failures could also reflect inappropriate trial populations. Typically, trials have recruited patients with mild to moderate dementia in whom the disease process may be too advanced to be amenable to treatment. An alternative approach is to treat subjects at risk of AD dementia, which is the aim of secondary prevention—preventing neurodegeneration, cognitive decline, and dementia [7, 8].

Research criteria from the International Working Group (IWG) [9, 10] and the National Institute on Ageing-Alzheimer Association (NIA-AA) [11–13] propose the use of biomarkers to define pre-clinical AD as the disease stage characterised by amyloid pathology, with or without neurofibrillary tangles and/or features of neurodegeneration, even in the absence of clinical manifestations. Mild cognitive impairment (MCI) is used to denote an intermediate stage between normal cognition and dementia in which subjects have objective cognitive impairment in the absence of functional disability interfering with daily activities. MCI with biomarker evidence of AD has been termed prodromal AD under the IWG criteria [9, 10] and MCI due to AD by the NIA-AA in 2011 [12], but the 2018 research framework does not take into account syndromic diagnosis [11]. Non-demented subjects with evidence of amyloid pathology, i.e. subjects with AD without dementia [14], provide an opportunity for intervention prior to irreversible neuronal loss. Designing trials for subjects in pre-dementia stages of AD is greatly facilitated by the ability to identify subjects at increased risk of cognitive decline and progression to dementia. In the earliest stage, neuropsychological testing to detect subtle cognitive abnormalities in the absence of obvious symptoms has some predictive value [15], but biomarkers that are sensitive to underlying pathological change could further increase prognostic accuracy. Ideally, early-stage biomarkers should predict risk and likely timing of cognitive decline and progression to dementia in a reliable, non-invasive, and cost-effective manner. Secondly, trials in non-demented subjects would benefit from biomarkers that can monitor treatment effects and should thus reflect disease progression, be sensitive to detect pathologically significant changes over time and in response to treatment, and be highly reproducible and reliable in a multi-centre setting. Neuroimaging techniques have developed rapidly over the past decade and they currently offer a comprehensive armamentarium that can be employed to address this unmet need. Over and above structural neuroimaging markers that are widely available and used to support AD diagnosis at the dementia stage, advances in imaging techniques allow the detection and quantification of molecular, functional, and structural brain changes that precede gross atrophy.

In this paper, we discuss the use of neuroimaging markers in subject selection for inclusion or stratification in secondary prevention trials, their potential to serve as outcome markers in trials, and for monitoring trial safety. The overall aim is to devise a neuroimaging strategy that will maximise the information required to enrol and monitor secondary prevention trials in AD (including progression from MCI to dementia). To identify potential imaging markers, a comprehensive review of the existing literature was performed. Based on the evidence from the literature and the personal experience of the authors, recommendations are centred around three domains: 1) imaging markers for subject selection and stratification; 2) imaging markers with potential to be used as outcome measures in clinical trials; and 3) imaging markers for monitoring trial safety. The utility of longitudinal imaging as a run-in for clinical trials, identification of exclusion criteria, and vascular comorbidity were also taken into account.

## Methods

### Neuroimaging modalities: from molecular changes to structural damage

**Fig. 1:**
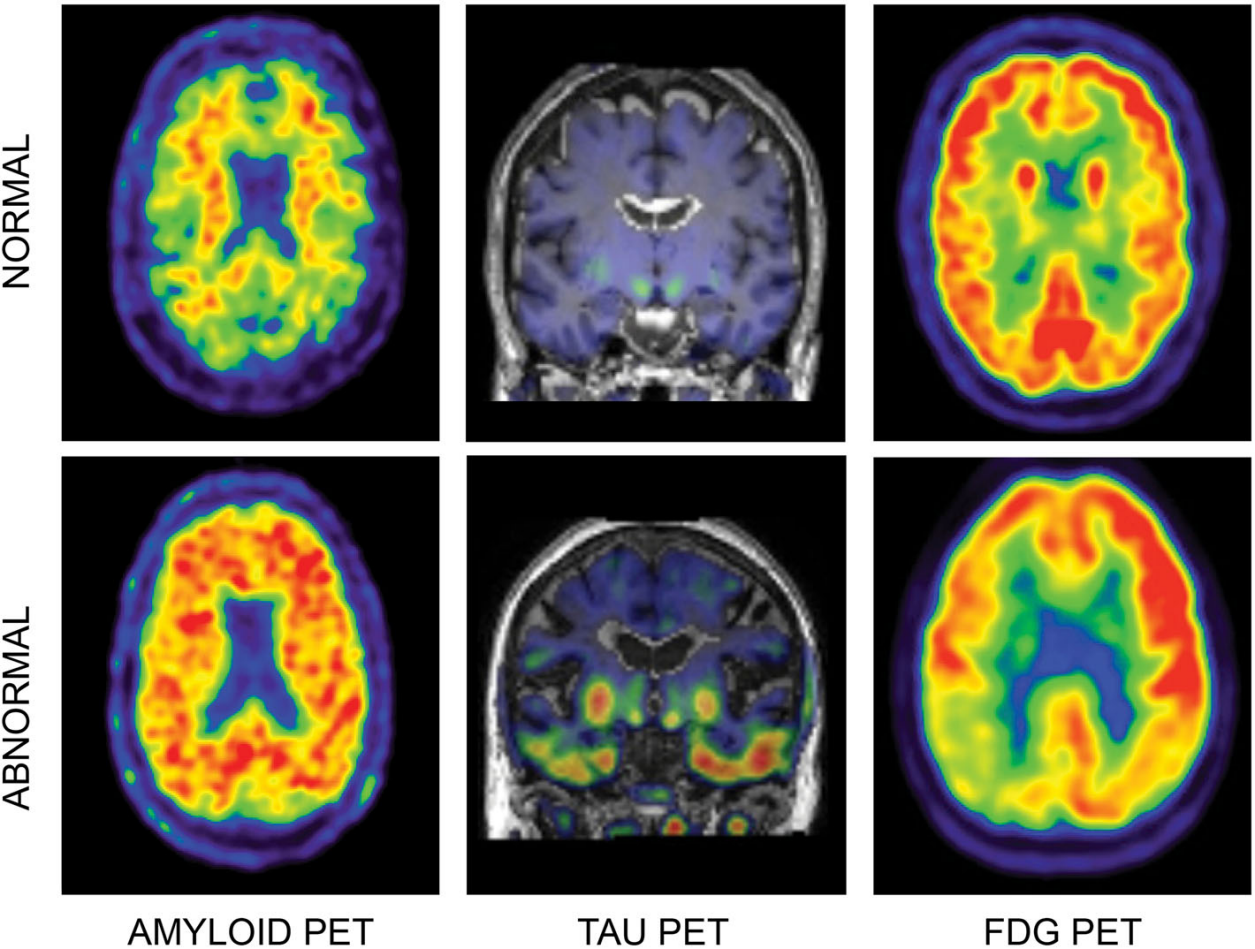
PET imaging biomarkers. Examples of normal (top) and abnormal (bottom) positron emission tomography (PET) imaging markers in three different subjects. For all images, the warmer the colour, the more tracer binding. Left: amyloid PET with [^18^F]-flutemetamol. In the abnormal scan, diffuse tracer binding to fibrillary amyloid can be observed. Middle: tau PET with [^18^F]-AV-1451. In the abnormal scan, tracer binding to tau can be observed in the temporal lobes. Right: Fluorodeoxyglucose (FDG)-PET scan. In the abnormal scan, there is hypometabolism of the parietal lobes

**Fig. 2:**
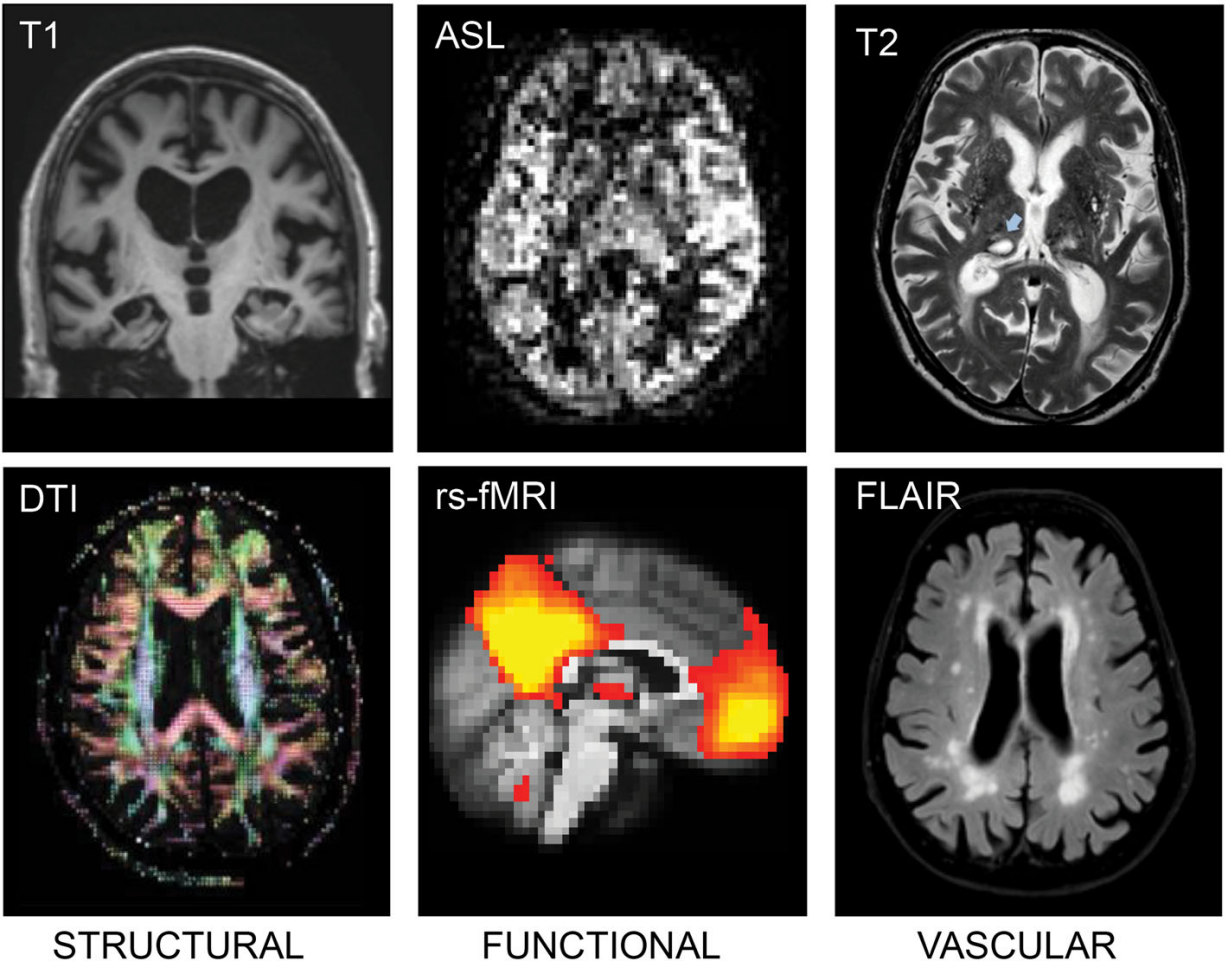
MRI imaging biomarkers. Left: T1-weighted MRI (top) showing severe hippocampal atrophy and example of diffusion tensor imaging (DTI) (bottom). Middle: example of functional imaging markers with arterial spin labelling (ASL) (top) and resting state functional magnetic resonance imaging (rs-fMRI) (bottom). Right: imaging of vascular pathology with thalamus lacune on T2 (top; arrow) and white matter hyper-intensities on fluid attenuated inversion recovery (FLAIR) (bottom)

The past two decades have seen major advances in neuroimaging. Different imaging modalities are now able to track different aspects of the hypothesised pathological cascade of events in AD in vivo [3] (Box 1, Figs. 1, and 2). Positron emission tomography (PET) with amyloid-specific tracers, and more recently also tau-binding ligands, can visualise and quantify molecular pathology at an early stage. Alterations in functional imaging biomarkers, reflecting early synaptic dysfunction and neuronal injury, can be measured with various PET and magnetic resonance imaging (MRI) measures. Collectively, molecular and functional changes may lead to synaptic loss, inflammation, white matter damage, and neuronal cell death, eventually leading to macroscopic changes such as regional and global brain atrophy seen on structural MRI. Increasing evidence demonstrates that cerebrovascular changes have an additive effect on neurodegeneration, accelerate cognitive decline and progression to dementia, and may even be part of the pathological cascade of AD [16–18]; hence, we also address MRI markers of vascular pathology.

### Search strategy and selection criteria

References for this review were identified by searching the PubMed/Medline database in August 2017. Relevant articles were identified using the following search terms alone and in varying combinations: “amyloid PET”, “tau PET”, “MRI”, “structural MRI”, “functional MRI”, “FDG PET”, “fluorodeoxyglucose PET”, “TSPO PET”, “diffusion tensor imaging”, “arterial spin labeling”, “magnetic resonance spectroscopy”, “cognitively healthy”, “normal cognition”, “mild cognitive impairment”, “subjective cognitive decline”, “Alzheimer’s disease”. Papers published in English were included. Further references were obtained by screening references from retrieved articles and on the basis of the personal knowledge of the authors. In the case of topics already extensively covered in the literature, as was often the case for MCI, a reference article or review was selected by the authors. The final selection of articles was based on relevance to the topics covered in this review, as judged by the authors

## Subject selection and stratification

Various planned and ongoing clinical trials for AD primarily target the amyloid cascade, aiming at the removal of amyloid plaques or prevention of misfolding of amyloid into the β conformation [19]. We recommend that trials targeting the amyloid pathway should include methods to recruit subjects with evidence of an appropriate level of amyloid pathology. Various PET tracers are capable of measuring and spatially localising β-amyloid deposits. Alternatively, β-amyloid peptides may be measured in cerebrospinal fluid (CSF). Studies have demonstrated good concordance between CSF β-amyloid 1–42 and amyloid PET measures, even though these markers might represent different pools of amyloid in the brain [20]. Several studies suggest that CSF β-amyloid levels become abnormal prior to an amyloid PET signal [21–23], making CSF markers more sensitive for the selection of subjects in the earliest phases of amyloid accumulation.

**Fig. 3:**
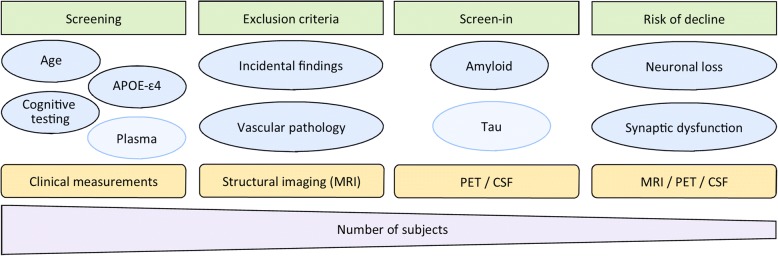
Step-wise approach for subject inclusion and testing. Information from clinical measurements (and, in the near future, possibly also plasma) may be used to select subjects with an increased risk of amyloid pathology (screening). Provided there are no exclusion criteria, molecular measurements of amyloid (or tau, depending on the treatment target) can be used to screen-in subjects for clinical trials. Finally, imaging measures predicting imminent cognitive decline may be used for additionally enrichment. APOE apolipoprotein E, CSF cerebrospinal fluid, MRI magnetic resonance imaging, PET positron emission tomography

The quantification of amyloid pathology with PET or in CSF for screening purposes in non-demented subjects is expensive, invasive, and likely inefficient. Therefore, a step-wise approach to subject inclusion is proposed (Fig. 3). Using demographic, genetic, and other clinical data, subjects may be screened for increased risk of amyloid pathology. In a recent study, the positive predictive value to detect amyloid-positive subjects at the pre-clinical stage, based on a combination of demographic information, apolipoprotein E (APOE) ɛ4 genotype, and neuropsychological changes over 24 months, was 0.65, which was 60% higher than the prevalence of amyloid in the cohort [24]. Moreover, plasma assays for β-amyloid have been recently developed, with preliminary evidence suggesting high performance in predicting cerebral amyloid burden [25, 26]. The potential scalability of this method, its cost-benefit ratio, and the minimal invasiveness make it a very promising tool in clinical trials, which could be incorporated early in the screening process. After screening for an increased probability of amyloid positivity, subjects may first undergo structural MRI which, combined with clinical information, can be used to exclude subjects with incidental findings that might interfere with the assessment of clinical outcomes or with a high likelihood of having or developing neurodegenerative diseases other than AD (Additional file 1: Table S1). Imaging changes associated with other neurodegenerative disorders are, however, unlikely to be present in the early stages, which may preclude the reliable exclusion of these subjects.

Structural and functional imaging markers can also be used to identify subjects at risk of imminent cognitive decline, which will be reviewed in the following paragraphs. This is especially relevant for phase 3 trials in subjects with pre-clinical AD targeting cognition as a primary outcome. Enrichment of clinical trials targeting clinical end-points by means of amyloid PET and/or MRI may reduce sample sizes and costs, as shown in subjects with MCI [27, 28], but this work needs to be extended to the pre-clinical phase. Table 1 summarises the available evidence for the use of different imaging markers for subject selection in clinical trials.

**Table 1:** Summary of evidence for use of imaging markers for subject selection and as outcome measures in clinical trials in pre-dementia Alzheimer’s disease

	Imaging technique	Pathological specificity for Alzheimer’s disease	Prediction of progression in cognitively normal	Prediction of progression in MCI	Reproducibility	Sensitivity to change	Response to treatment
Molecular	Amyloid PET	Strong	Moderate	Strong	Good	Moderate	Established
	Tau PET	Preliminary evidence with promising results	Unknown	Unknown	Preliminary evidence	Unknown	Unknown
Functional	ASL	Moderate	Weak	Weak	Moderate	Preliminary evidence	Preliminary evidence for exercise intervention
	rs-fMRI	Moderate	Unknown	Weak	Moderate	Preliminary evidence	Preliminary evidence for symptomatic drugs
	FDG-PET	Moderate	Moderate/good	Strong	Good	Good	Established for symptomatic drugs
Structural	T1: Hippocampal volume	Moderate	Good; although long follow-up is needed	Strong	Good	Good	Established, although few effective studies
	T1: Cortical atrophy	Moderate	Moderate/good depending on regions; long follow-up is needed	Good	Good	Good	Unknown
	DTI	Moderate	Weak	Moderate	Moderate	Unknown	Unknown

*AD* Alzheimer’s disease, *ASL* arterial spin labelling, *DTI* diffusion tensor imaging, *FDG* fluorodeoxyglucose, *MCI* mild cognitive impairment, PET positron emission tomography, *rs-fMRI* resting state functional magnetic resonance imaging

### Molecular imaging

#### Amyloid PET: predictor of decline?

Amyloid pathology measured with PET is an established prognostic marker in subjects with MCI (sensitivity 82% (95% confidence interval (CI) 74–88) and specificity 56% (95% CI 49–64) to distinguish stable MCI patients from those who progress to dementia) [29]. In cognitively normal subjects, amyloid positivity has been associated with an increased risk of cognitive decline and progression to dementia in several longitudinal studies [30–41], although studies with sufficiently long follow-up and large sets of data to establish the exact risk are required [40–43] (Table 2). The method of choice to classify subjects as amyloid-positive or amyloid-negative remains a matter of debate (Box 2). Recent evidence has also suggested that amyloid plaques might follow consistent deposition patterns in different regions of the brain, making it possible to stage amyloid pathology [44]. Although the relationship between amyloid positivity and later cognitive decline in cognitively normal subjects has been established, it has been suggested that the rate at which this occurs depends on the presence of neurodegeneration [45, 46]. Amyloid positivity is also consistently associated with increased brain atrophy rates in cognitively normal subjects (Additional file 1: Table S2). Hence, amyloid pathology is a necessary factor to assess whether an individual will decline due to AD pathology but is not sufficient to stage disease, or to predict when and how fast the decline will occur, since the timing depends on the rate of neurodegeneration [41, 46–48].

**Table 2:** Prediction of cognitive decline using amyloid PET in cognitively normal subjects

Reference	Study design				Tracer	Main outcome
	Cohort	Size	Follow-up	Mean age		
Donohue et al., 2017 [40]	ADNI	*N* = 445	Median 3.1 years	74	Various tracers or CSF	Aβ+: worse mean scores after 4 years on Preclinical Alzheimer Cognitive Composite score, MMSE and CDR-SB.
Petersen et al., 2016 [39]	Mayo Clinic Study of Aging	*N* = 564	Median 2.5 years	78	PiB	Aβ+: increased rate of cognitive decline in various cognitive domains and progression to MCI.
Vemuri et al., 2015 [38]	Mayo Clinic Study of Aging	*N* = 393	Mean 2.7 years	78	PiB	Aβ+: increased rate of cognitive decline compared to Aβ–.
Lim et al., 2012 [34]	AIBL	*N* = 141	18 months	76	PiB	Aβ+: greater cognitive decline on working memory and verbal and visual episodic memory.
Lim et al., 2014 [35, 73]	AIBL	*N* = 320	36 months	70	Various tracers	Aβ+: greater cognitive decline on verbal and visual episodic memory.
Rowe et al., 2013 [41]	AIBL	*N* = 183	36 months	72	PiB	Aβ+: predictor of progression to MCI/dementia (OR 4.8).
Kawas et al., 2013 [36]	90+ study	*N* = 13	Median 1.5 years	94	Florbetapir	Aβ+: steeper declines on most cognitive tests, particularly global cognitive measures.
Doraiswamy et al., 2014 [37]	AV45-A11 study. Multi-site, USA	*N* = 69	36 months	70	Florbetapir	Aβ+: greater decline on ADAS-Cog, digit-symbol-substitution test, verbal fluency test and CDR-SB.
Villemagne et al., 2011 [147]	Austin Health Memory Disorder Clinic and Melbourne Aging Study	*N* = 32	Mean 20 months	73	PiB	Aβ high: 16% conversion rate to MCI by 20 months Aβ high: 25% conversion rate to MCI by 3 years Aβ low: 1 subject developed MCI
Storandt et al., 2009 [31]	Washington University ADRC	*N* = 135	Up to 19 years. pre-PET	75	PiB	Increased cognitive decline in episodic and working memory in amyloid positive subjects (cognition measured before PET scan).
Morris et al., 2009 [30]	Washington University	*N* = 159	Mean 2.4 years	71.5	PiB	Higher mean cortical binding potential values predicted progression to AD (HR 4.85, 1.22–19.01).
Mormino et al., 2014 [46]	Harvard Aging Brain Study	*N* = 166	Median 2.1 years	74	PiB	Cognitive decline over time was observed only in cognitively healthy individuals who were Aβ+ and had evidence of neurodegeneration.
Resnick et al., 2010 [32]	Baltimore Longitudinal Study of Aging	*N* = 57	Mean 10.8 years	78.7	PiB	Aβ high: greater decline in mental status and verbal learning and memory, but not visual memory. Significant associations in frontal and lateral temporal regions.

*Aβ+/−* amyloid positive/negative, *AD* Alzheimer’s disease, *ADAS-cog* Alzheimer’s Disease Assessment Scale-cognitive subscale, *ADNI* Alzheimer’s Disease Neuroimaging Initiative, *ADRC* Alzheimer’s Disease Research Center, *AIBL* Australian Imaging, Biomarker and Lifestyle study, CDR-SB Clinical Dementia Rating sum of boxes, *CSF* cerebrospinal fluid, *HR* hazard ratio, *MCI* mild cognitive impairment, *MMSE* Mini-Mental State Examination, *OR* odds ratio, *PET* positron emission tomography, *PiB* Pittsburgh compound B

#### Tau PET: novel biomarker of neurofibrillary pathology

The deposition of neurofibrillary tangles of hyper-phosphorylated and aggregated tau proteins presents with an anatomical distribution mirroring that of neuronal loss and is more closely related in time and magnitude to clinical symptoms than amyloid pathology [3, 49, 50]. Tau PET tracers have been recently developed, and while clinical validation remains limited to date [51] the field is rapidly evolving. The development of tau tracers has been challenging given the intracellular localisation of tau, the relatively low concentrations, and multiple isoforms [51, 52]. The first tau tracers have off-target binding in the basal ganglia and choroid plexus [52]. More recently developed tracers might offer better technical characteristics, including the absence of intra-parenchymal off-target binding in these regions [53].

Since neurofibrillary tangles are more closely related to the onset of symptoms than amyloid, tau PET might be used in the future to identify subjects who are at elevated risk of imminent clinical decline. There are not yet any published prospective longitudinal studies on the predictive value of tau PET on cognitive decline, but it is expected that this area will evolve quickly in the next few years. One study has shown that increased tau-tracer binding in the entorhinal and hippocampal regions was associated with a worse prior longitudinal decline in global cognition and episodic memory measures [54]. Another study showed that, in the cognitively normal elderly, high amyloid and neocortical tau measured with PET was associated with posterior cingulate hypometabolism, which is turn was associated with future cognitive decline [55]. Tau PET may also be valuable as an inclusion criterion (screen-in) or as a means to segregate participants (e.g. on the basis of tau load or degree of spread) or as an outcome measure (prevention of tau spread or signal increase), especially for trials specifically targeting tau pathology.

### Functional imaging

#### Altered brain glucose metabolism: fluorodeoxyglucose-PET

Patients with AD dementia show characteristic patterns of glucose hypometabolism measured with fluorodeoxyglucose (FDG)-PET, which are also predictive of progression to dementia in subjects with MCI [29, 56, 57]. Moreover, FDG-PET demonstrates a high negative predictive value for progression to AD dementia in subjects with MCI [57]. In cognitively normal subjects, baseline lateral temporo-parietal and posterior cingulate-precuneus hypometabolism can predict clinical progression to MCI or dementia with accuracies in the range of 70–80% [58, 59]. Hence, FDG-PET has some potential to be used as a marker for trial inclusion or stratification, although changes in cognitively normal subjects are very subtle and thresholds for abnormality are not established.

#### Reduced cerebral blood flow: arterial spin labelling

Arterial spin labelling (ASL) can detect changes in cerebral blood flow in patients with MCI and AD dementia [60, 61]. Only two studies have investigated the predictive value of ASL measures on clinical progression. In subjects with MCI, baseline hypoperfusion in the right inferior parietal cortex, middle frontal cortex, and precuneus was associated with cognitive decline [62]. In cognitively normal subjects, reduced blood flow in the posterior cingulate cortex was associated with the development of subtle neuropsychological deficits, and discriminated subjects with cognitive decline from stable subjects with a sensitivity of 59% and a specificity of 65% [63]. More research is needed on the predictive value of ASL, as well as standardisation of processing streams and establishment of cut-offs for abnormality, before it can be used as an inclusion criterion in clinical trials.

Blood flow is closely coupled to brain glucose metabolism and hence ASL could potentially offer an MRI-based alternative to FDG-PET, although further research is needed [60, 64, 65]. The early frames of dynamically acquired amyloid PET images also provide information about cerebral blood flow [66]. These early frames also strongly correlate with the metabolic profiles obtained using FDG-PET [67–69]. Using the early frames of an amyloid PET as a functional measure, rather than a separate FDG-PET, avoids additional patient burden in terms of radiation or separate visits. However, it does require scanning immediately following tracer injection (Box 3).

#### Changes in functional connectivity: resting state functional MRI

Subjects with AD dementia may show decreased brain connectivity [70]. Evidence of early disruption of connectivity measures in the pre-clinical stage is emerging, although with conflicting results [71–75]. There is a paucity of evidence on the predictive value of resting state functional MRI (rs-fMRI) for clinical progression. Only studies in MCI have been performed, with mixed findings which may be due to differences in processing streams and end-points [76–79]. Although rs-fMRI has potential, larger studies are needed to identify sensitive rs-fMRI markers and to define normative values. At this stage, rs-fMRI is not recommended as an inclusion criterion for trials.

### Structural imaging

#### Medial temporal lobe atrophy: a role in the pre-clinical phase?

Structural MRI provides in-vivo measures of global and regional brain volumes, such as the hippocampus and entorhinal cortex. Medial temporal lobe atrophy is a consistent finding in patients with AD dementia and can aid in the prediction of time to clinical progression in amyloid-positive subjects with MCI [80, 81]. Population-based studies have shown that hippocampal volume and hippocampal atrophy rates can predict future cognitive decline in cognitively normal subjects during long follow-up periods, in the order of 5–10 years [82–84] (Table 3). Recent data showed that cognitively normal subjects with hippocampal atrophy and positive amyloid PET were at risk of cognitive decline, whereas subjects with low hippocampal volume but negative for amyloid pathology were not, over a median follow-up of 6 years [85]. Importantly, regional brain volumes derived from structural MRI vary with age, head size, and scanner type in the absence of known pathology [86]. Defining cut-offs for abnormality using cross-sectional volumes should be corrected for these confounds. Due to large inter-individual variation in cross-sectional measures of hippocampal volume, also within the diagnostic group [87], the intra-individual rate of hippocampal volume loss over time has attracted growing interest as this approach negates the effects of inter-individual variability. A meta-analysis found an average rate of atrophy per year of 4.7% (95% CI 3.9–5.4) in AD dementia and 1.4% (95% CI 0.5–2.3) in healthy ageing, resulting in an average 3% difference in atrophy per year [88]. These findings highlight the advantage of having a trial-readiness cohort in which longitudinal imaging data can be used to provide more accurate change measures to improve subject selection strategies for clinical trials.

**Table 3:** Predictive value of hippocampal measures for cognitive decline in cognitively normal subjects

Reference	Study design				Measurement type	Main outcome
	Cohort	Size	Follow-up	Mean age		
Burnham et al., 2016 [85]	AIBL	*N* = 573	6 years	73	Hippocampal volume	Subjects with low hippocampal volume and evidence of amyloid pathology showed faster cognitive decline compared with subjects with normal biomarkers. Subjects with only decreased hippocampal volume in the absence of amyloid pathology did not show significant decline compared to the normal biomarker group
den Heijer et al., 2010 [83]	Rotterdam study (population-based)	*N* = 518	8 years	73–79	Hippocampal atrophy rate	Hippocampal atrophy rates predict cognitive decline in healthy subjects (HR 1.6, 1.2–2.3).
den Heijer et al., 2006 [82]	Rotterdam study (population-based)	*N* = 511	6 years	73–79	Hippocampal volume	Hippocampal volume associated with risk of dementia (HR 3.0, 2.0–4.6).
Martin et al., 2010 [84]	University of Kentucky AD Centre	*N* = 71	5 years	78–84	Hippocampal and subregions volume; entorhinal cortex volume	Greater atrophy in hippocampus (head and body) and entorhinal cortex in subjects converting to MCI. AUC 0.87 for hippocampal head, 0.84 for hippocampal body, 0.79 for entorhinal cortex.
Stoub et al., 2005 [94]	Rush Alzheimer’s Disease Center (Chicago, USA)	*N* = 58 (CN and MCI together)	5 years	80	Hippocampal volume and atrophy rates; entorhinal cortex volume and atrophy rates	Baseline entorhinal and slope of decline were predictors for AD. Baseline hippocampal volume and atrophy rates were not (after controlling for entorhinal cortex).

*AD* Alzheimer’s disease, *AIBL* Australian Imaging, Biomarker and Lifestyle study, *AUC* area under the curve, *CN* cognitively normal, *MCI* mild cognitive impairment, *HR* hazard ratio

The European Medicines Agency has officially qualified hippocampal volume as an enrichment biomarker for inclusion in clinical trials in subjects with MCI [89], and subsequent work has demonstrated that selection of subjects with smaller hippocampi will likely reduce the overall cost of a trial in amnestic MCI [28]. This work requires extension to even earlier disease stages.

Within the hippocampus, different sub-fields can be distinguished using high-resolution MRI sequences which may provide additional information beyond hippocampal volume [90]. Several studies have shown that CA1 specifically, and to a lesser extent also subiculum atrophy, is present at a very early stage of AD since it could be detected in cognitively normal individuals that later developed MCI or AD dementia [84, 91–93].

Decreased entorhinal cortex volume may also be a predictor of cognitive decline [84, 94]. In a grouped sample of non-demented subjects (comprising cognitively normal and MCI subjects), baseline entorhinal cortex volume and rate of atrophy were predictors for dementia, whereas baseline hippocampal volume and atrophy rates were not when controlling for entorhinal cortex volume [94]. Similarly, a reduced volume of the anteromedial temporal cortex has been found to be associated with the development of MCI or AD dementia in cognitively normal subjects [95]. Although a measurable decrease in entorhinal cortex thickness may precede changes in hippocampal and amygdala volume by several years [96], measurement error will hamper implementation in large multi-centre studies.

#### Atrophy markers beyond the hippocampal region

Besides medial temporal atrophy, other brain regions that typically show atrophy in AD dementia patients may have (additional) predictive value for decline in early disease stages. Several unbiased whole-brain voxel-based studies have found an AD-like pattern of more atrophied regions (involving the temporal neocortex, posterior cingulate, and orbitofrontal cortex in addition to the medial temporal lobe) in cognitively normal subjects up to a decade before the onset of dementia [97, 98]. More recently, software to reliably quantify cortical thickness in regions throughout the brain has become widely available. Reduced cortical thickness in the entorhinal, inferior, and middle temporal structures, where tau neurofibrillary tangles are most prominent prior to the emergence of cognitive symptoms, were proposed as an AD signature of atrophy, and these regions were associated with cognitive decline in cognitively normal subjects [99]. By comparing cortical thickness in patients with AD dementia with cognitively normal subjects, another group also identified a set of so-called “AD-signature regions” comprising precuneus and other parietal and frontal regions in addition to the inferior and medial temporal cortex [100]. Atrophy in these AD-signature regions could aid in the prediction of cognitive decline in cognitively normal subjects [100], although the medial temporal lobe cortex thickness may be a stronger predictor of clinical progression [101]. Other groups employing similar strategies have also found that cortical thickness in varying regions can aid in predicting cognitive decline in cognitively normal subjects [102, 103]. A recent study showed that the rates of 3-year grey matter volume changes in areas of the episodic memory network mirrored accelerated decline in episodic memory ability in pre-clinical AD [104].

Medial temporal lobe atrophy is typical of AD and is commonly associated with an amnestic presentation. Atypical variants of AD also exist with different cognitive profiles (pre-dominance of non-memory symptoms) and variations in atrophy patterns [105]. The extent to which atrophy corresponding to these variants is detectable in pre-clinical phases of the disease remains unknown. Tracking atrophy in association with cortices in addition to medial temporal lobe structures may help in identifying or filtering for atypical subtypes of AD.

#### White matter tissue integrity: diffusion tensor imaging

Alterations to white matter microstructure as measured with diffusion tensor imaging (DTI) can be detected in patients with AD dementia [106, 107], and subtle changes might already be observed in the pre-clinical stage [108, 109]. In cognitively normal subjects, reductions in fractional anisotropy in the fornix [110] and precuneus [111] may predict progression to MCI. A study in elderly subjects with small vessel disease found an increased risk of dementia after 5-year follow-up associated with increased hippocampal mean diffusivity [112]. Studies in patients with MCI have shown that predictive values for cognitive decline are often higher (above 75%) in single-centre studies compared with multi-centre studies [113–117]. More research into standardisation of DTI acquisition and processing streams is necessary before DTI can be used for subject selection or stratification in multi-centre clinical trials.

### Neuroimaging specificity in the pre-dementia phase

#### Differential diagnosis

To use imaging biomarkers for trial enrichment in pre-dementia stages, they should ideally not only predict future cognitive decline but also be specific for the underlying disease that is being targeted. Molecular markers measuring protein aggregates in the brain may be the most specific for Alzheimer’s pathology. However, amyloid pathology can also occur in Lewy body dementia (DLB) [118], and at advancing age in the absence of imminent cognitive decline. Tau pathology is also seen in DLB and MAPT mutation carriers [119], traumatic brain injury [120], and normal ageing [121], although deposition patterns and binding of tau PET ligands may be different. Atrophy of the medial temporal lobe can be seen in various neurodegenerative diseases, as well as in vascular dementia [122–125], although the patterns of loss within the medial temporal lobe may help distinguish AD from other diseases [126]. Conversely, concordance between imaging markers and cognition may erroneously seem discordant, with more educated subjects showing preserved cognition in the presence of extensive brain pathology, a concept that has been termed cognitive reserve [127].

#### Combining neuroimaging and other biomarkers

The above-mentioned studies highlight that to date there is no single biomarker fully specific for predicting imminent cognitive decline due to AD in non-demented subjects. There is increasing interest in using more than one biomarker for enrichment, and in such circumstances these can be applied either sequentially or in combination [28, 128, 129]. A statistical model can be developed in which multiple (imaging and non-imaging) biomarkers, along with demographic variables, baseline cognitive status, and APOE ɛ4 carrier status, can be used to identify subjects at risk of clinical progression for inclusion in trials. Cost-effectiveness should also be assessed here in which the additional costs, time, and burden of acquiring additional biomarkers are weighed against the added predictive value. Some work has been done on this topic for trials in MCI [28, 29], but this needs to be extended to the pre-clinical phase.

### Vascular comorbidity: a partner in crime

#### Vascular pathology: a catalyst of cognitive decline

A plethora of vascular pathology can be detected with MRI [130]. White matter hyper-intensities on T2-weighted MRI and fluid attenuated inversion recovery (FLAIR) are considered to reflect small-vessel ischemic damage in the brain. Alternative mechanisms include blood-brain barrier dysfunction, failed interstitial fluid drainage, and processes causing ischaemic damage [130]. Even white matter that appears “normal” on FLAIR may show abnormalities on DTI and blood-brain barrier measures [131]. Regardless of the exact mechanism(s), white matter hyper-intensities represent tissue disruption and contribute to cognitive dysfunction independently of neurodegenerative mechanisms [16]. Population-based studies show a causal relationship between the extent of white matter hyper-intensities and the future risk of cognitive decline, including AD dementia [17]. White matter hyper-intensities can be assessed using visual rating scales [132, 133] or quantified using dedicated software.

Cerebral microbleeds are small areas of hemosiderin deposition that can be visualised as dot-like hypo-intensities on T2* and even better on (three-dimensional) susceptibility-weighted imaging (SWI) [130]. Microbleeds are thought to be caused by damage to small vessel walls due to hypertensive lipohyalinosis, fibrinoid necrosis, amyloid angiopathy, or a combination of these processes. These lesions increase with the burden of other small vessel disease features and are frequently detected in subjects with MCI and AD dementia [134]. In a population-based study, the presence of multiple microbleeds was associated with future decline on various cognitive domains and an increased risk of dementia, including the AD type [135]. Another study in cognitively normal subjects also demonstrated an association between the presence of multiple microbleeds and decline in executive functioning [136].

#### Vascular pathology in clinical trials: when to exclude?

When participants fulfil diagnostic criteria for vascular dementia [137], they are typically excluded from clinical trials in AD. To secure subjects with a high likelihood of Alzheimer-only pathology, many studies exclude subjects with varying amounts of vascular burden. The Alzheimer’s Disease Neuroimaging Initiative (ADNI), for example, excluded all subjects with infarcts and multiple or strategic lacunes, whereas in clinical practice many AD patients have a variable amount of vascular pathology, and vascular lesions may catalyse the expression of cognitive impairment [39]. Current practice in clinical trials varies and no consensus to date has been reached to evaluate cases of mixed pathology. Excluding all cerebrovascular pathology in trials may be prudent in early (phase 1 and 2) studies which aim to establish proof of principle for specific AD pathway interventions. In phase 3 trials, it has been argued that enrolled subjects should reflect normal clinical practice. A more realistic approach would be to exclude only those with severe cerebrovascular pathology and to look at effect modification by vascular comorbidity in a pre-defined subgroup analysis. The presence of cerebrovascular disease increases strongly with advancing age [17] and is thus a more likely comorbidity alongside amyloid and tau in elderly subjects. Young subjects with abnormal molecular markers may have more pure forms of AD pathology.

## Imaging as an outcome measure in trials

In secondary prevention trials targeting pre-dementia AD it may be challenging to meet clinical end-points (e.g. progression to dementia) with reasonable sample sizes and duration of treatment, especially in the pre-clinical phase [138]. Therefore, there is an urgent need for outcome markers that reflect slowing or prevention of disease progression. The choice of imaging marker to be used as the outcome may depend on the treatment target. An imaging measure as a (secondary) outcome in multi-centre trials needs to be highly reproducible, standardised across scanning platforms (instrument and tracer), sensitive to change over time, and able to detect treatment effects (whether target engagement, efficacy, or safety). From a practical perspective, imaging biomarkers should be operationally straightforward to deploy in multi-centre studies, with low costs compared with (less effective) alternatives. Available evidence on these technical features of the neuroimaging measures will be discussed in the next paragraphs and is summarised in Table 1.

For official surrogacy qualification by regulatory agencies, an imaging marker should also demonstrate an association with clinical progression, such that any effects of the treatment on the marker predict clinical benefit of the therapy [139]. Currently, there is not enough evidence for any imaging marker to qualify as a primary outcome measure in trials.

### Molecular imaging

#### Amyloid PET

For therapies targeting the amyloid cascade, amyloid PET provides an opportunity to assess target engagement. Rates of amyloid deposition on PET are in the order of 1–3% per year in cognitively normal subjects, with higher rates in subjects with increased amyloid loads at baseline and in APOE ɛ4 carriers [2, 33, 47, 140–143]. Some studies have suggested an inverted U-shaped curve of amyloid accumulation dependent on baseline amyloid loads, with the highest accumulation rates in subjects with intermediate (but above normal positivity thresholds) amyloid load at baseline and lowest rates in subjects without any amyloid or those with highest amyloid loads [2, 4, 140]. These potential non-linear rates should be considered when planning interventional trials as they may be relevant for sample size calculations and assessment of intervention effects. For example, subjects with high (plateau phase) amyloid burden will not further accumulate amyloid, which might make it more difficult to observe treatment-related effects [144].

There are limited published data on the variability and standardisation of amyloid PET in multi-centre studies [145], although practical experience is being gained in current phase 3 trials [146]. There is more knowledge and experience from FDG-PET studies, which have been used for a longer time. Standardisation by means of phantom scanning and local calibration of scanners is mandatory for interpretation of data from multi-centre settings. Procedural control at imaging sites must ensure consistent and adequate scanner quality assurance, subject preparation, and camera placement and acquisition and reconstruction parameters. Test-retest variability for ^18^F-based amyloid tracers is generally good (in the order of 1–5%) [147–149]. However, in longitudinal studies, pathological changes and ageing can present additional variability beyond the tracer and system variance. Intra-subject variability can be high due to factors such as progressive deterioration in the subject’s ability to remain still and managing subject movement during scan acquisition [145]. Moreover, changes in blood flow associated with disease progression or in response to therapy over the course of the study could confound measurements relying on the standardised uptake value ratio (SUVr) [150] (Box 3). Optimisation of reference regions, better management of technical factors, dynamic scanning, or correction for perfusion changes should reduce such within-subject variability and allow more accurate determination of change in brain amyloid burden. Subject-related factors such as patient movement and changes in blood flow are likely to be less prominent in trials in pre-clinical populations.

Several clinical trials have already used amyloid PET as an end-point in patients with mild to moderate AD dementia [146, 151]. Interestingly, in the recent phase 1 study of the monoclonal antibody aducanumab, clinical effects seemed to be more pronounced in subjects with greater reduction of amyloid plaques on PET, measured with SUVr [152]. It is not yet clear how much removal of amyloid is needed for a meaningful change in amyloid PET.

#### Tau PET

Tau PET tracers and data are emerging, but current implementation is limited based on costs, availability, standardisation, and specificity of tracers. Emerging data on novel tau tracers indicate that the signal-to-noise ratio of some tracers in AD is good and that the test-retest performance is robust (test-retest variability 1–4%) [153]. Preliminary data on longitudinal patterns of tau accumulation in symptomatic AD have recently been published, showing heterogeneous longitudinal changes between subjects [154]. Recent evidence in cognitively normal subjects suggests that rates of tau accumulation are in the order of 0.5% per year in amyloid-positive subjects, while there was no tau accumulation in the cognitively normal group without amyloid [155]. The use of tau PET as an outcome marker in trials will face similar challenges as amyloid PET, and may be of particular relevance in trials specifically targeting tau accumulation. Further development is encouraged, as therapies targeting tau pathology are entering clinical trials [156].

### Functional imaging

#### FDG-PET

Several studies have examined the potential of FDG-PET as an outcome marker in clinical trials. FDG-PET measures show good reproducibility across scanners and in multi-centre settings, provided there is prior harmonisation across sites [157]. Older studies have estimated that to measure a reduction of 25% in progression with a power of 80% in an MCI population, 800–1200 subjects are required per arm [158]. When optimising acquisition procedures and analysis methods in multi-centre studies, the number of subjects needed decreases considerably, to about 200 subjects per arm [158, 159]. No sample size estimates are available yet for pre-clinical populations. FDG-PET has already been used as imaging end-point in several clinical trials in subjects with AD dementia [151], but with the advent of amyloid tracers has become less attractive as multiple (fluorinated) PET tracers would increase the radiation dose. Early-frame dynamic amyloid PET and ASL provide possible alternatives [60, 64, 67–69].

#### Arterial spin labelling

Some studies have started to use ASL as secondary outcome measures in trials investigating the effects of physical exercise on cognitive decline, but these have not yet been published (e.g. PACE-2 study [160]). Cerebral blood flow might be affected by the administration of therapeutical agents, but the power of ASL in detecting these changes still remains to be assessed. Quantifying cerebral blood flow from ASL techniques is not trivial since the signal-to-noise ratio is generally low and the measurement is influenced by various factors such as bolus arrival time, arterial input function, underlying kinetics, and static tissue parameters such as blood equilibrium magnetisation, in addition to subject factors such as age and disease. These underlying physiological variables may also themselves be altered in AD. In single-centre and single-vendor studies, the reproducibility of ASL measures is high [161, 162] but the differences between implementations of ASL sequences by scanner vendors are substantial [163]. Guidelines for the performance of ASL in clinical settings have been established [164] but are yet to be validated in multi-centre trials. Together with recent research focusing on standardising ASL measures across vendors [165], this will facilitate the use of ASL in multi-centre settings in the future. Nevertheless, ASL measures remain extremely sensitive to small changes in acquisition parameters, rendering it difficult at present to use in longitudinal settings where software updates may result in such parameter changes [165].

#### Resting state functional MRI

Test-retest reliability and across-scanner reproducibility of resting state functional networks have been established [166]. Recent large-scale studies have started to address multi-site harmonisation issues for longitudinal studies [167, 168]. Despite efforts to standardise the method, fMRI is susceptible to different sources of variability including physiological noise, head movement, and analytical methods which require further optimisation [169]. Due to harmonisation issues in longitudinal and multi-centre settings, we suggest that rs-fMRI measures can at present only serve as an exploratory outcome measure in trials. rs-fMRI has already been used as a secondary outcome measure in clinical trials in subjects with AD dementia, and treatment-related effects were found on the default mode network over a 3- to 6-month time period [170, 171]. rs-fMRI is currently also being used in multi-centre clinical trials in cognitively normal subjects at increased risk of AD (e.g. the A4 trial [8]).

### Structural imaging

#### Grey matter atrophy

Patients with AD dementia show a pattern of widespread cerebral atrophy. Measures of global cortical atrophy have been used as an outcome marker in clinical trials in MCI and AD dementia (e.g. [172–174]). Besides global measures, regional changes can also be examined. Regional changes in different brain regions will likely relate more or less strongly to changes in different cognitive instruments, depending on the cognitive domains they subserve [175].

Hippocampal atrophy rates are a good candidate to serve as an outcome marker in multi-centre clinical trials, as long as standardisation of image acquisition between centres, consistent within-subject scanner acquisition, and uniform post-processing methods are performed. Test-retest reliability of repeated manual and automated hippocampal measurements from the same scanner is usually high (test-retest variability 1–4%) [176, 177], but some algorithms are more robust than others [178]. Efforts to standardise the measurement of hippocampal volumetry in multi-centre studies have been undertaken and it is now standard practice for trials to employ single algorithms and centralised analysis [89, 179]. With the establishment of the Harmonised Protocol for hippocampal segmentation, there is a new gold standard against which automated measurement may be validated [180]. Variability between scanners using the same acquisition protocol and measurement algorithm is low [176]. However, the agreement in terms of absolute volumes varies with acquisition protocols and field strength. For example, change in voxel size can lead to systematic errors in the range of 5% for hippocampal volume [181]. Methods to correct for these variabilities are being investigated [86].

Using data from the Australian Imaging, Biomarker and Lifestyle (AIBL) study, a sample size of 384 subjects per arm was estimated to be needed to detect 25% slowing of hippocampal atrophy rates over 18 months in subjects with pre-clinical AD with a power of 80% [182]. These numbers will be larger when taking into account atrophy occurring with normal ageing, which should be considered [183]. Measures of hippocampal atrophy rates have already been used as (secondary) end-points in various clinical trials in MCI and mild AD dementia, with varying results [151].

#### Diffusion tensor imaging

To date, there is little evidence for the use of DTI measures in (multi-centre) clinical trials. Several studies have pointed to the variability of DTI measurements in multi-centre studies and the need for extensive site harmonisation and calibration prior to starting [184–186]. More work is needed on the optimisation of DTI measurements in multi-centre settings, which is now being addressed [187]. So far, longitudinal DTI measures have only sparsely been used as (secondary) outcomes in clinical trials [188].

## Monitoring of side-effects

### Amyloid-related imaging abnormalities

**Fig. 4:**
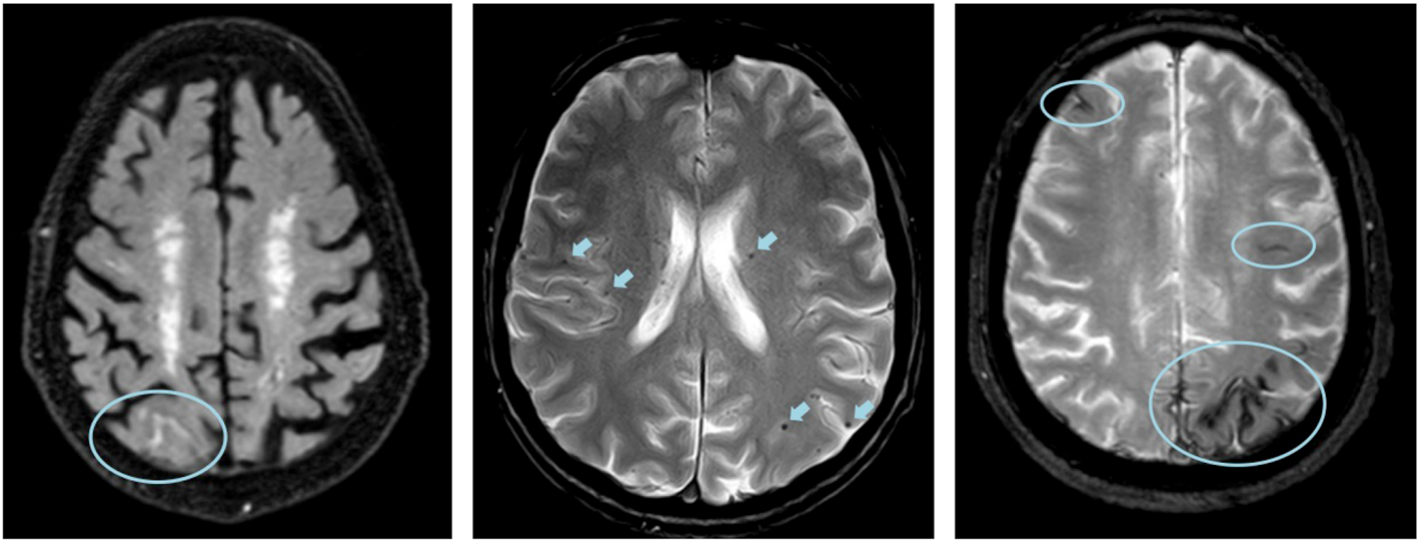
Amyloid-related imaging abnormalities. Example of ARIA-E on FLAIR with sulcal effusion (left) and ARIA-H with multiple microbleeds (middle) and superficial siderosis (right) on T2* images

MRI scans play an important role in safety monitoring during clinical trials. An issue of particular importance in the Alzheimer’s field is the occurrence of amyloid-related imaging abnormalities (ARIA) that have emerged in several anti-amyloid immunotherapy trials [189], although ARIA also occurs spontaneously—including in placebo arms [190]. ARIA consists of ARIA-E (parenchymal oedema or sulcal effusion) with signal hyper-intensities on FLAIR, and ARIA-H (cerebral microbleeds or superficial siderosis) with hypo-intensity due to hemosiderin on T2*-weighted or susceptibility-sensitive pulse sequences (Fig. 4). Adequate training for radiological reads is recommended for both ARIA-E and ARIA-H to ensure reliable detection of subtle cases and to maximise consistency between raters [191]. Severity of ARIA-E can be rated using dedicated rating scales [192]. Central assessment of the images is recommended to guarantee quality control and to minimise differences in visual inspection and quantification.

The detection of ARIA-H is dependent on the pulse sequence contrast mechanism and parameters (e.g. echo time and slice thickness) as well as field strength [189]. The reported prevalence of microbleeds in AD dementia subjects ranges from 18% at 1 T to 71% at 7 T [193]. Since the presence of cerebral microbleeds confers a risk for future haemorrhage and development of ARIA, many trials limit the number of microbleeds at entry and monitor development of new microbleeds during the trial. A key consideration in the management of emergent ARIA cases during a trial is whether any clinical symptoms are associated with the radiological observations.

In pre-clinical AD populations, the baseline incidence and the frequency of spontaneous emergent ARIA is less well characterised than in clinically demented subjects, although one study observed a lower incidence than in MCI and AD dementia populations [194]. Although several large randomised clinical trials in pre-clinical AD populations have recently commenced, the ARIA findings are not yet available to the wider community.

### Regulatory perspective

In 2011, following observations in the bapineuzumab trials [195] and recommendations from an Alzheimer’s Association Round Table workgroup [189], the US Food and Drug Administration (FDA) requested the adoption of ARIA-based exclusion and discontinuation criteria for amyloid-targeting therapies. These limited the enrolment to subjects with at most four microbleeds at baseline and defined minimum acquisition standards. Since that time, for non-amyloid targeting mechanisms or if the ARIA risk for a particular compound or mechanism has been discharged in earlier trials, these criteria have been relaxed in some trials. Nevertheless, these recommendations have become a de facto standard. Systematic data collection and comparison between 2D-T2* and more sensitive sequences (e.g. 3D susceptibility-weighted imaging) is lacking. A well-controlled head-to-head comparison of the above T2* sequence prescription with a more sensitive alternative sequence would further the field’s understanding of ARIA prevalence and evolution in the natural history and provide data to help establish meaningful and comparable cut-off criteria for these alternative sequences early in the disease course.

## Conclusions and practical implementation

Neuroimaging allows visualisation of many aspects of the pathological cascade of AD, including the presence of (pre-clinical) molecular pathology and downstream functional and structural markers of neurodegeneration before the onset of dementia. Information derived from imaging can aid in identifying non-demented subjects with an increased risk of future cognitive decline and disease progression to be included in secondary prevention trials. Combining information on underlying Alzheimer’s pathology from amyloid PET (or CSF) with markers of neurodegeneration from structural MRI (or FDG-PET) provides the optimal strategy to identify subjects who are at highest risk of cognitive decline within the typical time frame of a clinical trial. Advanced functional and structural imaging techniques to predict cognitive decline at a pre-dementia stage hold promise but await further research and validation. Neuroimaging can help to characterise subjects in terms of comorbidities (e.g. cerebrovascular disease) or to define more homogeneous subgroups that can be used for stratification. Neuroimaging can also serve as a (secondary) outcome marker in trials. Amyloid PET can be used to assess target engagement for pharmacological compounds targeting the amyloid cascade, and structural imaging (or FDG-PET) can be used to assess possible treatment effects on neurodegeneration. Finally, imaging is crucial for monitoring safety and potential side effects, such as ARIA, during trials.

Trials in non-demented subjects are greatly facilitated by the establishment of a cohort of well-phenotyped subjects that can be included in clinical trials. This is an approach that has been taken by the European Prevention for Alzheimer’s Dementia (EPAD; http://ep-ad.org/) study [196]. Within EPAD, subjects at elevated risk for AD are identified from various parent cohorts throughout Europe and enrolled into a longitudinal observational cohort study to serve as a trial-readiness cohort for proof-of-concept intervention studies. In addition to screening subjects, this trial-readiness cohort also provides an opportunity to apply run-in designs in which pre-trial longitudinal imaging can be used to determine within-subject rates of change, which increases the statistical power to detect treatment effects and reduces required sample sizes [197].

There are no formal guidelines on the use of neuroimaging measures in such trial-readiness cohorts of non-demented subjects. Some experience has been gathered through longitudinal imaging from multi-centre studies with large cohorts such as ADNI, with a strong focus on the MCI and dementia stages, and more recently with the AIBL study, with a focus on cognitively normal subjects [198, 199]. In ADNI, the imaging protocol initially included structural MRI and a subgroup with amyloid PET, and was later complemented by FDG-PET and advanced MR techniques such as ASL, DTI, and rs-fMRI in ADNI-2. Subjects in ADNI-3 also undergo tau PET. In AIBL, the imaging protocol includes structural MRI and amyloid PET. Based on the literature reviewed in this paper, experience gained in other studies, and practical considerations, neuroimaging recommendations for the EPAD longitudinal cohort study have been formulated (Table 4). These recommendations are based on a combination of desired information richness, patient burden, stratification capabilities, and provision of run-in data for trials. The recommended core imaging protocol for the EPAD longitudinal cohort study consists of yearly MRI scans including isotropic 3D-T1 and 3D-FLAIR sequences, as well as a short 2D-T2W and 2D-T2* sequence to assess neurodegenerative and vascular pathology. Advanced sequences such as 3D-T2*/SWI, DTI, ASL, and rs-fMRI will be acquired in subsets of patients depending on site interest and experience with acquisition methodology. Centralised assessment of the scans will be performed to guarantee quality control and maximise consistency in visual rating scales and quantification. All subjects in EPAD will undergo lumbar puncture to assess amyloid pathology in CSF. A large subgroup will also undergo amyloid PET, financed through the sister project Amyloid Imaging to Prevent AD (AMYPAD; http://amypad.eu/). Amyloid PET was prioritised as molecular imaging based on its potential to confirm and localise amyloid pathology, its broad availability and standardisation, and given that the initial molecular targets in upcoming proof-of-concept trials will likely address the amyloid pathway. Static amyloid PET imaging is currently the most common approach in clinical trials. Dynamic imaging, however, may be preferable to determine the true binding potential rather than an SUVr that may be biased by the choice of the reference region and flow effects. This argument becomes more relevant for longitudinal imaging where changes can be small and in treatment trials where flow alterations may occur. In addition, data from the initial uptake of the tracer can be used as a proxy to measure cerebral blood flow. As FDG-PET would add an additional radiation dose, it has not been included in the EPAD imaging protocol. While tau PET imaging is emerging, current implementation is limited due to costs, availability, and lack of standardisation. Development and accessibility of tau PET is encouraged as a potentially more proximate biomarker and predictor of disease progression and as therapies targeting tau enter clinical trials.

**Table 4:** Imaging recommendations for EPAD longitudinal cohort study

Imaging technique	Baseline	Baseline use	Follow-up	Follow-up use	Measures
3D T1	Standard	Exclusion criteria Volumetric analysis Visual rating pathology Identify increased risk of decline	Annually	New incidental findings Atrophy rates Track visual pathology Identify increased risk of decline	Volumetric analysis: brain structures Visual: lacunes, atrophy
3D FLAIR	Standard	Exclusion criteria Volumetric analysis Visual rating pathology Subject stratification	Annually	New incidental findings Track visual pathology	Volumetric analysis: white matter hyperintensities Visual: lacunes, white matter hyperintensities, ARIA
2D-T2	Standard	Exclusion criteria Visual rating pathology	Annually	New incidental findings Track visual pathology	Visual: lacunes, perivascular spaces, ARIA
2D-T2* 3D-T2*/ SWI	Standard Optional	Exclusion criteria Visual rating pathology	Annually	New incidental findings Track visual pathology	Visual: microbleeds and superficial siderosis
DTI	Optional	Exploratory analysis	Optional 2-year	Changes in measures	Axial and radial diffusivity
ASL	Optional	Exploratory analysis	Optional 2-year	Changes in measures	Whole brain and regional perfusion
rs-fMRI	Optional	Exploratory analysis	Optional 2-year	Changes in measures	Connectivity measures
Amyloid PET (static and dynamic)	Optional	Inclusion criteria	Optional	Changes in measures	Visual: amyloid positive Quantitative measures of amyloid pathology Dynamic scanning: perfusion measures

*ARIA* Amyloid-related imaging abnormalities, *ASL* arterial spin labelling, *DTI* diffusion tensor imaging, *EPAD* European Prevention for Alzheimer’s Dementia, *FLAIR* fluid attenuated inversion recovery, *PET* positron emission tomography, *rs-fMRI* resting state functional magnetic resonance imaging

## Box 1: Neuroimaging armamentarium

***Molecular markers***

Amyloid PET is a reliable in-vivo marker of β-amyloid plaque load. The first amyloid-specific tracer, ^11^C Pittsburgh compound B (PiB), was developed in 2004 and binds with high affinity to fibrillary amyloid [200]. With the introduction of ^18^F-labelled amyloid tracers (florbetapir, florbetaben, flutemetamol) which obviate the need for on-site cyclotron and radiochemistry facilities, amyloid PET has become more widely available [201]. Recently developed tau PET tracers have entered clinical testing and might be used as a measure of tau pathology [51]. Tau PET imaging recapitulates Braak stages in vivo, making tau PET a possible measure of neurofibrillary pathology spread [54, 202].

***Functional markers***

PET imaging with ^18^F-fluorodeoxyglucose (FDG-PET) measures cerebral glucose metabolism and is used in clinical practice for the (differential) diagnosis of neurodegenerative disorders. Patients with AD typically show hypo-metabolism in the posterior cingulate and temporo-parietal cortices [56]. Arterial spin labelling (ASL) is a non-invasive MRI technique for quantification of cerebral blood flow. Cerebral blood flow is coupled to brain metabolism, and reduced blood flow patterns may overlap with those of glucose hypo-metabolism [60, 64]. Resting state functional MRI (rs-fMRI) measures spontaneous brain function by examining inter-regional coupling of low-frequency temporal oscillations in the blood oxygenation level-dependent (BOLD) signal. Intrinsic brain networks in AD mainly show decreased functional connectivity in the default mode network [70].

***Structural markers***

Medial temporal atrophy visualised on MRI is the most established imaging marker for AD at the dementia and MCI stages [203]. Using T1-weighted MRI, volumetric measures of structures such as the hippocampus and entorhinal cortex can be reliably obtained by automated segmentation. With higher resolution proton density or T2 MRI, hippocampal subfields can be delineated to further specify the location of atrophy [90]. Diffusion tensor imaging (DTI) detects the diffusion of water molecules in neural tissue reflecting white matter integrity [204]. In subjects with AD, DTI abnormalities can be detected in the posterior regions, limbic structures, and corpus callosum [106, 107].

## Box 2: cut-offs for imaging biomarker abnormality

Subject selection on the basis of imaging markers requires the definition of criteria for inclusion. These may involve visual reads and/or pre-defined thresholds for quantitative measures to classify markers as either normal or abnormal. In clinical practice, some imaging markers are commonly interpreted by visual inspection (e.g. atrophy on MRI or amyloid PET), but (semi-)quantitative methods have been suggested to provide more sensitive readouts [5]. The definition of cut-off scores for quantitative imaging markers remains a topic of active research and is dependent on the type of marker, the acquisition method, its intended use, and the relative requirement to maximise sensitivity or specificity [205, 206]. A frequently applied strategy for quantitative markers involves taking the 10th or 90th percentile (depending on the imaging biomarker) of either a normal reference population or an AD dementia cohort. Alternatively, thresholds may be based on an optimal separation of cognitively normal subjects from dementia patients [206].

In recent studies on ageing, lower thresholds for amyloid pathology than those used to separate cognitively normal from AD dementia patients have been proposed [5, 41]. While screening subjects for inclusion in clinical trials, it might be beneficial to also identify subjects with sub-threshold amyloid deposition, as many might have progressed to supra-threshold levels and be in the phase of rapid amyloid accumulation by the time an intervention study commences [4, 140]. This would allow the best window of opportunity for secondary prevention. It may be valuable to stratify sub-populations based on their amyloid burden into those clearly negative for amyloid pathology, sub-threshold amyloid pathology, and clearly abnormal amyloid burden, rather than dichotomise them into negative/positive categories. It has been suggested that staging of amyloid pathology might be possible, but further investigation is required to assess the feasibility of this [44].

## Box 3: Quantification of amyloid load from PET

Quantification of amyloid load from PET images can be performed with different types of measurements. The most commonly used parameter is the integrated signal in target-rich cortical regions compared with a target-poor region such as the cerebellum, subcortical white matter or pons, also referred to as the standardised uptake value ratio (SUVr). SUVr can be derived from static scans, typically a 20-minute acquisition 1 h after injection, when uptake of the tracer has plateaued. The choice of target regions of interest and reference regions for normalisation has led to a wide range of published SUVr cut-off values for subject amyloid status classification. Amyloid load can also be quantified more specifically with binding potential values or distribution volume ratios, which are derived from kinetic modelling. This requires a longer acquisition time, with dynamic scanning from the moment of tracer injection until the plateau phase. Due to the lower variability, lower sensitivity to flow, and ability to measure subtle longitudinal changes, fully quantitative measurements may be preferred over SUVr [150, 207]. Further research is needed to establish whether dynamic, rather than static, amyloid PET imaging is operationally practical and cost-effective for determining true binding potential in early proof-of-concept trials aiming to slow the rate of amyloid accumulation.

## Additional file

Additional file 1: **Table S1.** Incidental findings on MRI. **Table S2.** Effect of amyloid on longitudinal MRI measures in cognitively normal subjects. (PDF 107 kb)

## Abbreviations

AD: Alzheimer’s disease
AMYPAD: Amyloid Imaging to Prevent Alzheimer’s Disease
APOE: Apolipoprotein E
ARIA: Amyloid-related imaging abnormalities
ASL: Arterial spin labelling
CI: Confidence interval
CSF: Cerebrospinal fluid
DLB: Lewy body dementia
DTI: Diffusion tensor imaging
EPAD: European Prevention for Alzheimer’s Dementia
FDG: Fluorodeoxyglucose
FLAIR: Fluid attenuated inversion recovery
MCI: Mild cognitive impairment
MRI: Magnetic resonance imaging
PET: Positron emission tomography
PiB: Pittsburgh compound B
rs-fMRI: Resting state functional magnetic resonance imaging
SUVr: Standardised uptake value ratio
