# Supplementary material for: Secondary prevention of Alzheimer’s dementia: neuroimaging contributions
Source: Alzheimers Res Ther. 2018 Oct 30;10:112. doi: 10.1186/s13195-018-0438-z (PMC6208183; doi:10.1186/s13195-018-0438-z)
Supplement: Supplementary file 1 — Table S1. Incidental findings on MRI. Table S2. Effect of amyloid on longitudinal MRI measures in cognitively normal subjects. (PDF 107 kb) [file 13195_2018_438_MOESM1_ESM.pdf]

Secondary prevention of Alzheimer's dementia - neuroimaging contributions

Mara ten Kate, Silvia Ingala, Adam J. Schwarz, Nick C. Fox, Gaël Chételat, Bart N.M. van Berckel, Michael Ewers, Christopher Foley, Juan Domingo Gispert, Derek Hill, Michael C. Irizarry, Adriaan A. Lammertsma, José Luis Molinuevo, Craig Ritchie, Philip Scheltens, Mark E. Schmidt, Pieter Jelle Visser, Adam Waldman, Joanna Wardlaw, Sven Haller, Frederik Barkhof

Supplementary Tables

Table S1: Incidental findings on MRI that may interfere with assessment of clinical and/or cognitive outcomes

**Clinically significant findings (reasons for exclusion)**
Subdural hematoma with compression
Territorial infarcts
Recent parenchymal haemorrhage
Obstructive hydrocephalus
Malignant intra-axial tumours (gliomas, metastases)
Large extra-axial tumours
**Potentially significant findings**
Clear evidence of a neurodegenerative disorder other than AD
Extensive deep white matter lesions (Fazekas grade 3)
Multiple lacunes (>4)
Small chronic subdural hematoma or effusion without compression
Small benign extra-axial tumours without mass effect
Communicating hydrocephalus
Aneurysms, AVM or cavernoma
Large arachnoid cysts with compression
Carotid occlusion
Cerebral microbleeds with evidence of past macroscopic haemorrhage
Demyelinating disease
Significant osseous and extra-cranial soft tissue pathology

Table S2: Effect of amyloid on longitudinal MRI measures in cognitively normal subjects.

Reference	Study Design				Measurement type	Main outcome
	Cohort	Size	Follow-up	Mean age		
**Burnham 2016**	AIBL	N=573	6 years	73	- PiB-PET<br>- HC volume<br>- HC atrophy rate	Subjects with abnormal amyloid and low hippocampal volume at baseline had faster hippocampal atrophy rates than those with normal biomarkers at baseline.
**Schott 2010**	ADNI	N=105	1 year	75	- CSF amyloid<br>- HC atrophy rate<br>- Ventr atrophy rate<br>- WB atrophy rate	Aβ+: Increased atrophy rates (all measures).
**Andrews 2013**	AIBL	N=66	18 months	73	- PiB-PET<br>- HC atrophy rate<br>- Ventr atrophy rate<br>- WB atrophy rate	Aβ+: Increased hippocampal atrophy rates. Non-significant higher rates of whole brain and ventricular atrophy.
**Doré 2013**	AIBL	N=93	36 months	74	- PiB-PET<br>- Cortical thickness	Aβ+: Increased atrophy rates in temporal lobe and hippocampus. Reduced cortical thickness in precuneus and hippocampus associated with episodic memory impairment.
**Nosheny 2014**	ADNI	N=566 including MCI	4 years	75	- PiB / AV45 PET<br>- HC atrophy rates	Aβ+: Increased hippocampal atrophy rates.
**Mattsson 2014**	ADNI	N=47	Up to 4 years	76	- ROI volumes<br>- Cortical thickness<br>- CSF amyloid	Aβ+: Increased atrophy rates in frontoparietal regions, cingulate, amygdala and temporal regions.
**Chetelat 2012**	AIBL	N=74	18 months	74	- PiB-PET	Aβ+: Higher rates of atrophy; highest significance in temporal neocortex and posterior cingulate cortex.
**Sabuncu 2011**	ADNI	N=92	1 year	67	- CSF amyloid<br>- HC volume<br>- HC atrophy rate<br>- AD-regions cortical thickness	Aβ+: Reduced baseline cortical thickness. No difference in hippocampal volume at baseline or atrophy rates.

**Jack 2014**	Mayo Clinic Study of Aging	N=252	Median 1.4 years	78	- PiB-PET<br>- AD-signature region atrophy rate	Increased brain atrophy rates in subjects with both Aβ+ and evidence of neurodegeneration (decreased hippocampal volume) at baseline
**Knopman 2013**	Mayo Clinic Study of Aging	N=191	15 months	77	- PiB-PET<br>- HC atrophy rates	Increased hippocampal atrophy rates in subjects with both Aβ+ and evidence of neurodegeneration (hippocampal atrophy or FDG-PET hypometabolism) at baseline.
**Desikan 2011**	ADNI	N=107	Mean 2.14 year	76	- CSF<br>- Entorhinal cortex atrophy	Increased entorhinal cortex atrophy only in subjects with both decreased CSF Aβ and evidence of neurodegeneration (elevated CSF phosphorylated tau) at baseline.
**Ewers 2012**	ADNI	N=124	2 years	76	- PiB-PET	Aβ+: Increased medial temporal lobe and precuneus volume decline
**Petersen 2016**	Mayo Clinic Study of Aging	N = 286	Median 2.5 y	78	- PiB-PET<br>- HC atrophy rates<br>- Ventricular enlargement	Increased hippocampal atrophy and ventricular enlargement rates associated with elevated amyloid levels.
